# Association of Social Media Presence with Online Physician Ratings and Surgical Volume Among California Urologists: Observational Study

**DOI:** 10.2196/10195

**Published:** 2019-08-13

**Authors:** Justin Houman, James Weinberger, Ashley Caron, Alex Hannemann, Michael Zaliznyak, Devin Patel, Ariel Moradzadeh, Timothy J Daskivich

**Affiliations:** 1 Cedars-Sinai Medical Center Los Angeles, CA United States; 2 David Geffen School of Medicine University of California Los Angeles Los Angeles, CA United States

**Keywords:** social media, surgical volume, physician ratings

## Abstract

**Background:**

Urologists are increasingly using various forms of social media to promote their professional practice and attract patients. Currently, the association of social media on a urologists’ practice is unknown.

**Objectives:**

We aimed to determine whether social media presence is associated with higher online physician ratings and surgical volume among California urologists.

**Methods:**

We sampled 195 California urologists who were rated on the ProPublica Surgeon Scorecard website. We obtained information on professional use of online social media (Facebook, Instagram, Twitter, blog, and YouTube) in 2014 and defined social media presence as a binary variable (yes/no) for use of an individual platform or any platform. We collected data on online physician ratings across websites (Yelp, Healthgrades, Vitals, RateMD, and UCompareHealthcare) and calculated the mean physician ratings across all websites as an average weighted by the number of reviews. We then collected data on surgical volume for radical prostatectomy from the ProPublica Surgeon Scorecard website. We used multivariable linear regression to determine the association of social media presence with physician ratings and surgical volume.

**Results:**

Among our sample of 195 urologists, 62 (32%) were active on some form of social media. Social media presence on any platform was associated with a slightly higher mean physician rating (β coefficient: .3; 95% CI 0.03-0.5; *P*=.05). However, only YouTube was associated with higher physician ratings (β coefficient: .3; 95% CI 0.2-0.5; *P*=.04). Social media presence on YouTube was strongly associated with increased radical prostatectomy volume (β coefficient: 7.4; 95% CI 0.3-14.5; *P*=.04). Social media presence on any platform was associated with increased radical prostatectomy volume (β coefficient: 7.1; 95% CI –0.7 to 14.2; *P*=.05).

**Conclusions:**

Urologists’ use of social media, especially YouTube, is associated with a modest increase in physician ratings and prostatectomy volume. Although a majority of urologists are not currently active on social media, patients may be more inclined to endorse and choose subspecialist urologists who post videos of their surgical technique.

## Introduction

Social media use is becoming increasingly common among both health care consumers and urologists. A recent Pew Research Center study showed that the number of US internet users active on social media has increased from 8% in 2005 to 74% in 2014 [1]. Urologists are also part of this trend; in 2014, more than 70% of urologists were reported to be active on some form of social media [2,3]. Urologists currently use social media for a variety of reasons, including discussing patient cases, sharing patient education materials, creating forums to discuss journal articles, and connecting attendees at large academic conferences [4,5]. Beside these reasons, there are numerous academic advantages to social media use to expand professional networks, create new opportunities for academic collaboration, and increase citation potential for papers [6]. However, another presumed intent of social media among physicians is improvement in business productivity in terms of case volume and public perception. Physicians who are active on social media are undoubtedly trying to promote themselves or their practice. Steinmetz et al [7] highlighted how various forms of advertising, such as social media, can improve sales, profits, and reputation of a business.

Despite this, to date, there has been no published literature on the impact of social media on the public perception of providers or business productivity.

Although surgical volume is a clear indicator of business productivity, public perception is a more intangible concept that is difficult to measure, especially given the lack of published literature in this area. However, online physician ratings may be a reasonable proxy for the public’s perception of a physician, based on the scope of use and impact of online ratings on health care consumers’ behavior. This is evidenced by the fact that in the United States, 47% of patients performed online searches of their physicians in 2010 [8], and a recent population-based analysis of 600 physicians showed a median of seven reviews per provider [9]. In 2010, one in six practicing US physicians had received an online review [10]. These ratings are frequently used as a key source of information through which patients choose a physician. This is further supported by a survey of 1000 surgical patients at the Mayo clinic, which found that 81% of patients would seek consultation from a physician based on positive reviews alone, and 77% would not seek consultation from a physician based solely on negative reviews [11]. Similar data have been reported in Europe [12]. Because of the strong association of online ratings with health care consumers’ choices, it may be reasonable to use online physician ratings as a proxy measure of reputation, if this is defined as the likelihood that a patient will choose to consult with a given practice based on community opinions.

In this study, we aimed to determine whether social media presence among urologists impacts their reputation (vis-à-vis their online consumer rating) and surgical volume. To address this question, we sampled 195 California urologists rated on the ProPublica Surgeon Scorecard website to determine whether professional use of social media platforms (Facebook, Instagram, Twitter, blog, and YouTube) was associated with average numeric physician rating across five popular websites and radical prostatectomy surgical volume in 2014 as determined by the Medicare Physician and Other Supplier Public Use File. Recognizing the potential for confounding by institutional branding and practice setting, we corrected for whether the urologist was affiliated with an academic or private practice and performed a subgroup analysis to determine if the effect size was consistent.

We hypothesized that physicians with a more active social media presence would have higher online physicians ratings and surgical volume.

## Methods

### Data Source and Participants

The Cedars-Sinai Institutional Review Board provided an exemption certification for this study (IRB #00050328)**.** This study was conducted in accordance with all relevant guidelines and procedures of Cedars-Sinai Medical Center. We sampled all California urologists rated on the ProPublica Surgeon Scorecard website (n=195). The ProPublica Surgeon Scorecard website was used because it provided the most comprehensive list of currently practicing urologist. These urologists completed at least 20 radical prostatectomy or transurethral resection of the prostate procedures in the calendar year 2014 according to Medicare claims data [13]. Physicians were excluded from the online review portion of the analysis if they had no online reviews (n=12). Physicians were excluded from the surgical volume portion of the analysis if they performed less than 20 radical prostatectomies in the calendar year 2014 (n=110).

### Variables

#### Primary Predictor

The primary predictor was physician social media presence. One of the investigators (JH) collected data on physicians’ professional social media presence in calendar year 2014 on five popular social media platforms: Facebook, Instagram, Twitter, YouTube, and professional blog. Social media presence was considered a binary variable (yes/no), defined as any social media posts promoting their medical practice. We coded social media presence both at the individual platform level and across all platforms. We also collected data on frequency of posts, but elected not to subdivide our sample based on this characteristic given the uniformity of frequency (90% posted information on social media once a month, and only 10% posted information with greater or lesser frequency).

#### Covariates

We gathered demographic data on physicians in our sample, including practice setting (academic or private), years since medical school graduation, and location of medical school (domestic or international) from the California Medical Board website.

#### Outcomes

##### Online Physician Ratings

We collected online ratings for each physician across the 5 most popular online physician-rating platforms according to Google Trends: HealthGrades, Vitals, Yelp, RateMD, and UCompareHealth. Each of these websites asks consumers to rate physicians using a “5-star” scale. Using these data, we calculated each physician’s “average rating” as a weighted average of scores across the five websites, weighted by the number of reviews on each website.

##### Physician Surgical Volume

We collected data on radical prostatectomy surgical volume in 2014 using Medicare claims from the Medicare Physician and Other Supplier Public Use File. We linked these data to other data sources using National Physician Identifier numbers. We defined radical prostatectomy as Current Procedural Terminology claims codes 55840, 55842, and 55845.

### Statistical Analysis

We compared characteristics of our sample population by activity on social media, using the Chi-square test for categorical variables and the Wilcoxon-Mann-Whitney test for nonparametrically distributed dependent variables.

We used multivariable linear regression analysis to assess the association of physician social media presence with online physician ratings. Our primary predictor in these models was social media presence, and the outcome was average online physician rating across the five websites. Covariates included practice setting, years since medical school graduation, and location of medical school. We created separate models to analyze the impact of social media presence on any platform and individual social media platforms on online physician ratings. We also performed sensitivity analyses of our aggregate model in subgroups of academic and private physicians.

We assessed the association of physician social media presence on physician surgical volume in a similar fashion, using multivariable linear regression analysis and identical predictor and covariate structure. We created separate models to analyze the impact of social media presence on any platform and of individual social media platforms on radical prostatectomy volume. We also performed sensitivity analyses of our aggregate model in subgroups of academic and private physicians.

## Results

### Overview

Characteristics of our sample across those active on social media versus those not active were recorded. Of the 195 California urologists, 62 (32%) were active professionally on some form of social media in 2014, including 53 (27%) on YouTube, 15 (8%) on Facebook, 14 (7%) on Twitter, 10 (5%) on blogs, and 6 (3%) on Instagram. Of the total, 159 urologists were in the private practice setting and 36 were in the academic setting. The average number of years since medical school graduation was 32.6 years. In addition, 163 attended medical school in the United States and 32 attended foreign medical schools (Multimedia Appendix 1).

### Association of Social Media Presence With Online Physician Ratings

Multivariable linear regression models predicting the weighted average of online physician ratings showed that social media presence on any platform was associated with a significantly higher mean physician rating (β coefficient: .3; 95% CI 0.03-0.5; *P*=.05; Multimedia Appendix 2). A similar magnitude and direction of the effect persisted among subgroups of private (β coefficient: .2; 95% CI –0.1 to 0.5; *P*=.2) and academic physicians (β coefficient: .6; 95% CI 0.15-1.0; *P*=.01) in sensitivity analyses. However, in multivariable models assessing the association of individual social media platforms and online ratings, only presence on YouTube was associated with a significantly higher mean physician rating (β coefficient: .3; 95% CI 0.2-0.5; *P*=.04). There were no meaningful or statistically significant differences in online physician ratings with regard to the use of other social media platforms.

### Association of Social Media Presence With Physician Surgical Volume

Multivariable linear regression models predicting surgical volume showed that social media presence on any platform was associated with a trend toward higher annual radical prostatectomy volume (β coefficient: 7.1; 95% CI –0.7 to 14.2; *P*=.05; Multimedia Appendix 3). A similar magnitude of effect was observed among subgroups of private (β coefficient: 7.1; 95% CI –0.9 to 15.2; *P*=.08) and academic (β coefficient: 6.7; 95% CI –11.3 to 24.8; *P*=.4) physicians in sensitivity analyses. In multivariable models assessing the association of individual social media platforms with surgical volume, presence on YouTube was significantly associated with higher annual radical prostatectomy volume (β coefficient: 7.4; 95% CI 0.3-14.5; *P*=.04; Multimedia Appendix 3). There was no statistically significant difference in surgical volume with regard to the use of other social media platforms.

## Discussion

Although most California urologists were not active on social media, we found that professional use of social media was associated with higher online physician ratings and increased prostatectomy volume.

Although physicians are increasingly using online social media to interact with their patients and promote their practices, to date, it is unclear whether this activity has any demonstrable effect on productivity outcomes. Our study suggests that professional activity on social media sites may positively impact both the physician’s reputation as well as their surgical volume. We found that social media activity on any of the five social media outlets studied (and YouTube specifically in subgroup analysis) had a statistically significant association with online physician ratings, with an average increase of 0.3 over physicians who were not active on social media. Although a 0.3 increase on a 5-point scale seems small, it represents a difference of 0.4 SDs from the mean, indicating a meaningful difference. We also found that presence on any one of the five social media sites was associated with a trend toward increased prostatectomy volume, with a statistically significant increase noted among those active on You Tube. A urologist who posts videos on social media is likely to perform roughly seven more radical prostatectomies per year than a urologist who does not post videos (representing an average increase of roughly 25% over the mean annual rate of 27 prostatectomies).

To our knowledge, this is the first study to directly test the association between social media use and clinical productivity outcomes. Other surgical specialties have speculated on the advantages of social media use in clinical efficiency and productivity afforded by social media outreach, but have not engaged in formal hypothesis testing. Orthopedic surgeons have postulated that directing patients to social media sites focused on patient education may enable more efficient and effective communications with their patients, reducing patient phone call volume and increasing clinical efficiency [14]. Vascular surgeons have suggested the utility of social media outreach in circumventing traditional physician referral patterns, providing an advantage in gaining market presence [15]. Despite clear interest in the association between social media use and real-world outcomes, there remains a lack of evidence-based testing of these associations. Our study, while limited due to its retrospective design, provides some evidence for the purported utility of professional use of social media.

Our finding that YouTube is the social media form with the strongest association with a physician’s online reputation and surgical volume is consistent with recent data showing that health care consumers can accurately identify quality of surgery by watching online samples of a surgeon’s technique. A recent study showed that medically trained reviewers were able to identify surgeons with higher complication rates by watching videos of their technique in the context of laparoscopic bariatric surgery [16]. Surprisingly, health care consumers (via crowdsourcing among the general population) were also able to identify surgeons with higher complication rates by watching videos of their operative technique in the context of robotic radical prostatectomy [17]. Based on this finding, patients may be justified in choosing surgeons who post videos online, since they appear to be able discern good from bad surgeons by viewing examples of their best work.

Professional use of social media is one of a number of sources of information that contribute to a physician’s “online dossier,” and both health care consumers and physicians should be aware of the relative worth of each component. The comprehensive online data available to health care consumers for choosing a physician include social media activity, online physician ratings, quality metrics, surgical volume, office ratings, and publicly available personal information. Although this study highlights the importance of social media presence in cultivating an online persona, the other components undoubtedly can (and should) contribute to a patient’s overall perception of a physician. For example, we recently argued that online rating data should not be used as the sole criterion to select physicians by health care consumers (as data suggests they are), since they have no association with quality or value of care [18]. Similar to online ratings, social media presence should not be used by patients in isolation to select physicians. Ideally, physicians and patients will consider social media activity in the context of other sources of information that provide independent information about the physician, such as data that capture quality of care (surgical volume, quality metrics, and complexity of case volume), value of care, and the patient experience (online ratings).

This study has several limitations that may affect our findings. First, the association of social media presence with reputation and productivity outcomes may be confounded by academic institutional branding. However, the magnitude of association was virtually identical for subsets of private and academic physicians, which suggests that the findings are robust across different practice settings. Second, we are unable to rule out reverse causality as an explanation for our findings (ie, whether being a high-volume, highly rated surgeon is predictive of activity on social media). However, even if some degree of reverse causality exists, the policy implication is still the same: More surgeons should be posting videos online to prove the adequacy of their skills compared to their peers. Finally, the observational nature of this study may incur selection bias, since it only includes California urologists who are performing a minimum of 20 urologic procedures (transurethral resection of the prostate and radical prostatectomy) per year. Considerations for future prospective study design could include an interrupted time series or a randomized controlled study.

In conclusion, our findings suggest that urologists should consider being active on social media to promote and build their professional practice. Given the trajectory of use of online resources such as online ratings in selecting providers, we believe that the use of social media will become an increasingly important outreach tool for clinicians to interact with their patients in a meaningful way.

## Appendix

Multimedia Appendix 1 Sample characteristics (n=195).

Multimedia Appendix 2 Association between social media presence and online physician ratings.

Multimedia Appendix 3 Association between social media presence and prostatectomy volume.
