# Supplementary material for: Association of Social Media Presence with Online Physician Ratings and Surgical Volume Among California Urologists: Observational Study
Source: J Med Internet Res. 2019 Aug 13;21(8):e10195. doi: 10.2196/10195 (PMC6711043; doi:10.2196/10195)
Supplement: Multimedia Appendix 1 [file jmir_v21i8e10195_app1.pdf]

Table 1: Sample Characteristics (n=195)

Variable	Active on Social Media (n=62)	Not Active on Social Media (n=133)	P-Value
**Practice Setting**			<0.001
Academic	21 (34%)	10 (8%)	
Private Practice	41 (66%)	123 (92%)	
**Location of Medical School**			0.2
International	7 (11%)	25 (19%)	
Domestic	55 (89%)	108 (81%)	
**Median Years since Medical School Graduation (IQR)**	25 (18, 37)	36 (24, 45)	0.002
**Median Number of Reviews (IQR)**			
Healthgrades	11 (8, 17)	10 (5, 14)	0.03
RateMD	3 (1, 5)	3 (1, 6)	0.7
UCompareHealthcare	4 (2, 8)	4 (1.5, 6)	0.5
Vitals	6 (3, 14)	5.5 (2, 12)	0.3
Yelp	4 (2, 5)	3 (2, 5)	0.4
