# Supplementary material for: Association of Social Media Presence with Online Physician Ratings and Surgical Volume Among California Urologists: Observational Study
Source: J Med Internet Res. 2019 Aug 13;21(8):e10195. doi: 10.2196/10195 (PMC6711043; doi:10.2196/10195)
Supplement: Multimedia Appendix 2 [file jmir_v21i8e10195_app2.pdf]

Table 2: Association between social media presence and online physician ratings

Social Media Platform	Difference in Mean Physician Rating Score (vs. No Social Media Activity) (95% Confidence Interval)	P-Value
Facebook	0.0005 [-0.4 – 0.4]	0.5
Instagram	0.08 [-0.5 – 0.7]	0.6
Blog	0.15 [-0.3 – 0.6]	0.3
Twitter	0.04 [-0.4 – 0.5]	0.3
YouTube	0.3 [0.2–0.5]	0.04
Any Social Media Platform	0.3 [0.03 – 0.5]	0.05
