# Supplementary material for: Association of Social Media Presence with Online Physician Ratings and Surgical Volume Among California Urologists: Observational Study
Source: J Med Internet Res. 2019 Aug 13;21(8):e10195. doi: 10.2196/10195 (PMC6711043; doi:10.2196/10195)
Supplement: Multimedia Appendix 3 [file jmir_v21i8e10195_app3.pdf]

Table 3: Association between social media presence and prostatectomy volume

Social Media Platform	Change in Prostatectomy Volume (95% Confidence Interval)	P-Value
Facebook	-2.7 (-14.0 – 8.6)	0.6
Instagram	7.8 (-9.7 – 25.2)	0.4
Blog	7.4 (-3.9 – 18.6)	0.2
Twitter	5.6 (-5.2 – 16.4)	0.3
YouTube	7.4 (0.3 – 14.5)	0.04
Any Social Media Platform	7.1 (-.07 – 14.2)	0.05
